# CBCT-guided Caldwell–Luc management of a dentigerous cyst associated with an ectopic maxillary third molar in the maxillary sinus: a case report

**DOI:** 10.3389/fdmed.2026.1891853

**Published:** 2026-08-04

**Authors:** José Luis Llanos-Arteaga, Daniel Andrés Delgado-Piedra, Luis Alberto Llanos-Arteaga, Anderson Daniel Llanos-Arteaga, Juan Marcos Parise-Vasco

**Affiliations:** 1 Posgrado de Cirugía Oral, Universidad Central del Ecuador, Quito, Ecuador; 2 Cirugía Maxilofacial, Hospital De Especialidades Eugenio Espejo, Quito, Ecuador; 3 Estomatología, Ministerio de Salud Pública, San Miguel De Bolívar, Ecuador; 4 Center for Evidence Ecosystems, Implementation Science, and Decision-Making (CIDES), Facultad de Ciencias de la Salud y Bienestar Humano, Universidad Tecnológica Indoamérica. Ambato, Ecuador

**Keywords:** dentigerous cyst, odontogenic cysts, maxillary sinus, Caldwell–Luc procedure, healthcare, quality of life, case report

## Abstract

A dentigerous cyst associated with an ectopic third molar in the maxillary sinus is an extremely rare pathological entity that poses diagnostic and therapeutic challenges. We report the case of a 22-year-old woman with a four-month history of chronic nasal congestion and postnasal drip sensation, without a relevant medical history. Cone-beam computed tomography (CBCT) confirmed a cystic lesion associated with an ectopic third molar, allowing accurate three-dimensional assessment for surgical planning, with partial involvement of the left maxillary antrum. Given the size and location of the lesion, complete cystic capsule enucleation and ectopic tooth odontectomy were performed using the Caldwell–Luc approach under local anesthesia. Histopathological examination confirmed the diagnosis of a dentigerous cyst. Clinical and imaging follow-up at six months showed complete resolution of sinonasal symptoms and no evidence of recurrence. This case highlights that the Caldwell–Luc approach guided by CBCT remains a viable and effective option for treating extensive odontogenic cystic lesions involving the maxillary sinus.

## 1 Introduction

A dentigerous cyst is one of the most common odontogenic lesions of epithelial origin and develops from the reduced enamel epithelium. It is associated with unerupted or impacted teeth and accounts for approximately 20% of all jaw cysts, representing the second most prevalent odontogenic lesion after the radicular cyst (1). Ectopic tooth eruption is an uncommon condition in which a tooth erupts in an abnormal anatomical site, such as the coronoid process, mandibular condyle, nasal cavity, or, exceptionally, the maxillary sinus. However, it is often asymptomatic; it may present with pain, recurrent infections, and bony expansion (2). The association between ectopic eruption and dentigerous cysts is an even rarer finding, documented in approximately 1% of reported cases (2). The clinical presentation ranges from incidental findings on routine imaging studies to symptoms such as nasal obstruction, chronic sinusitis, facial pain, purulent discharge, or orbital complications secondary to cystic expansion (1, 3).

The diagnosis of these lesions requires systematic clinical, imaging, and histological correlation (4). Orthopantomography is usually the initial detection tool; however, computed tomography (CT) and cone-beam computed tomography (CBCT) provide an accurate three-dimensional assessment of cyst extension, the location of the ectopic tooth, and its relationship with adjacent structures, all of which are essential for surgical planning (2, 5). Magnetic resonance imaging (MRI) can also be considered as a complementary imaging modality for maxillary sinus lesions; however, its use is limited in routine dental practice due to cost and availability (6, 7). More recent literature emphasizes the increasing role of cone-beam computed tomography in improving diagnostic accuracy and surgical planning in odontogenic lesions involving the maxillary sinus (6).

The treatment of choice is surgical enucleation of the cyst along with removal of the ectopic tooth. The classic approach for lesions in the maxillary sinus has been the Caldwell–Luc technique, which provides wide, direct access to the sinus cavity, facilitating complete removal of the lesion (7). However, this procedure may be associated with complications such as infraorbital hypoesthesia, the development of an oroantral fistula, or alterations in sinus function (2). In recent years, transnasal functional endoscopic sinus surgery has gained increasing relevance as a minimally invasive alternative, offering advantages such as reduced tissue trauma, lower morbidity, and faster recovery (8). Nevertheless, its applicability depends on the precise location of the lesion and the position of the ectopic tooth, since in large lesions or cases with difficult anatomical access, the Caldwell–Luc approach remains the most viable option (9, 10).

Given the rarity of this clinical presentation and the ongoing controversy surrounding the optimal surgical approach, its detailed description is of clinical and scientific relevance. This case contributes to the literature by highlighting CBCT-guided surgical planning, complete enucleation using the Caldwell–Luc approach under local anesthesia, and favorable short-term clinical and imaging outcomes in a resource-limited setting. The objective of this case report is to describe the diagnosis, CBCT-guided planning, and surgical treatment of a dentigerous cyst associated with an ectopic left upper third molar in the maxillary sinus using the Caldwell–Luc approach.

## 2 Case report

A 22-year-old woman presented to the Department of Oral and Maxillofacial Surgery with a chronic four-month history of nasal congestion and a sensation of postnasal drip. The patient did not present a relevant personal, family or surgical medical history and denied alcohol consumption, smoking, or substance abuse. The extraoral evaluation showed preserved facial symmetry. Intraoral examination revealed normal oral mucosa, no signs of bony expansion, and clinical absence of the upper left third molar of the dental arch.

**Figure 1:**
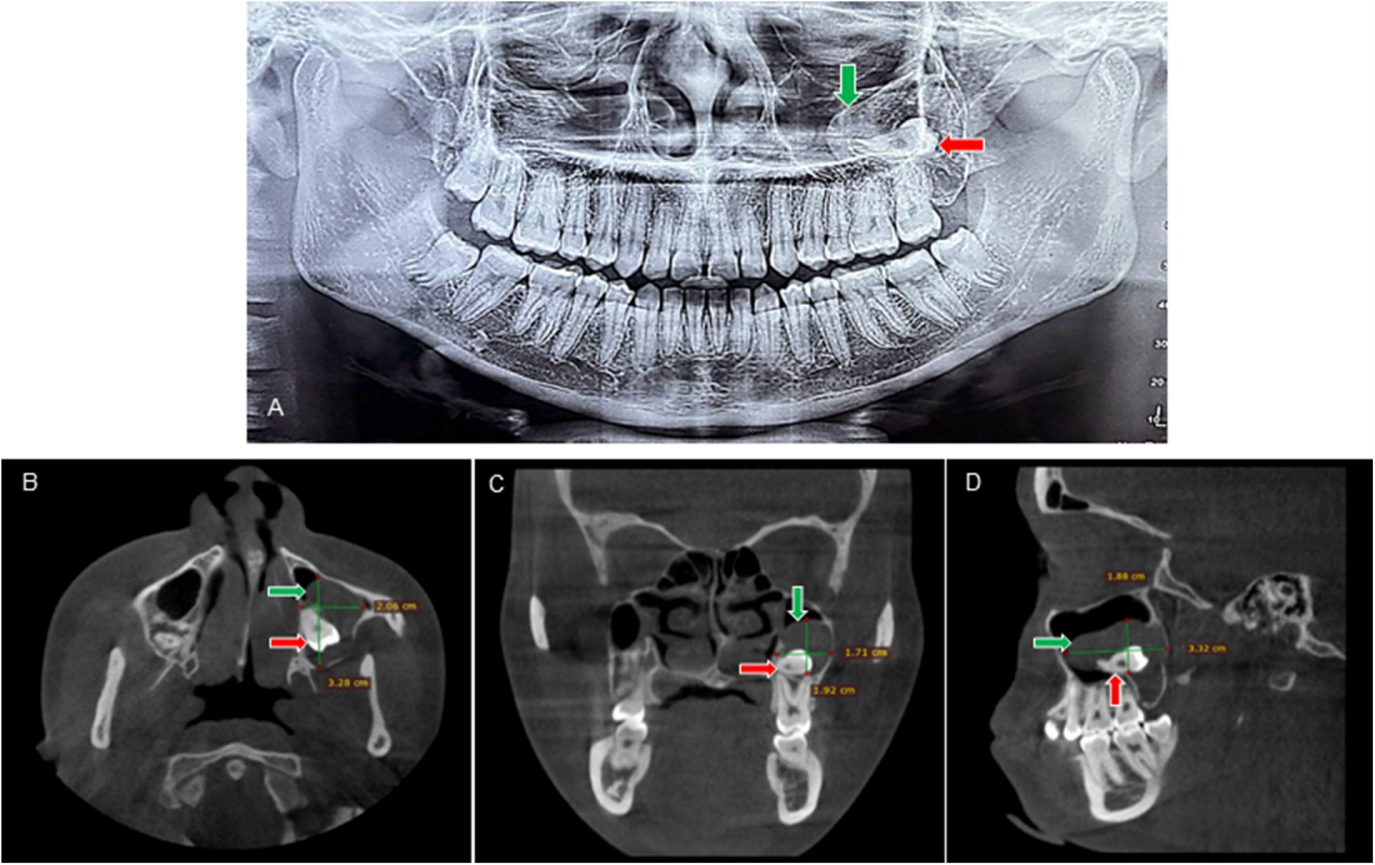
**(A)** preoperative orthopantomography: a radiolucent image with a central radiopaque focus compatible with an ectopic tooth in the left maxillary sinus is observed. Cone-beam computed tomography (CBCT): **(B)** axial, **(C)** coronal, and **(D)** sagittal views showing the ectopic left maxillary third molar associated with a well-defined hypodense image compatible with a cystic lesion. Red arrows indicate the ectopic left maxillary third molar, and green arrows indicate the margins of the associated cystic lesion.

Orthopantomography revealed a radiolucent image associated with the crown and root of the left third maxillary molar, which was displaced to the ipsilateral maxillary sinus. CBCT was performed to confirm the extent and location of the lesion (Figure 1). It demonstrated a hypodense unilocular image with well-defined borders, measuring approximately 3.3 × 2.0 × 1.9 cm (anteroposterior   ×   mediolateral   ×   superoinferior; greatest dimension ≈ 3.3 cm), associated with the ectopic left maxillary third molar, leading to cortical expansion, bony wall thinning, and partial occupation of the left maxillary sinus. These imaging characteristics allowed the differential diagnosis to be narrowed to a dentigerous cyst, an odontogenic keratocyst, or a unicystic ameloblastoma. Once the exact location of the lesion had been confirmed, complete cystic capsule enucleation was planned together with the ectopic left maxillary third molar using the Caldwell–Luc technique.

**Figure 2:**
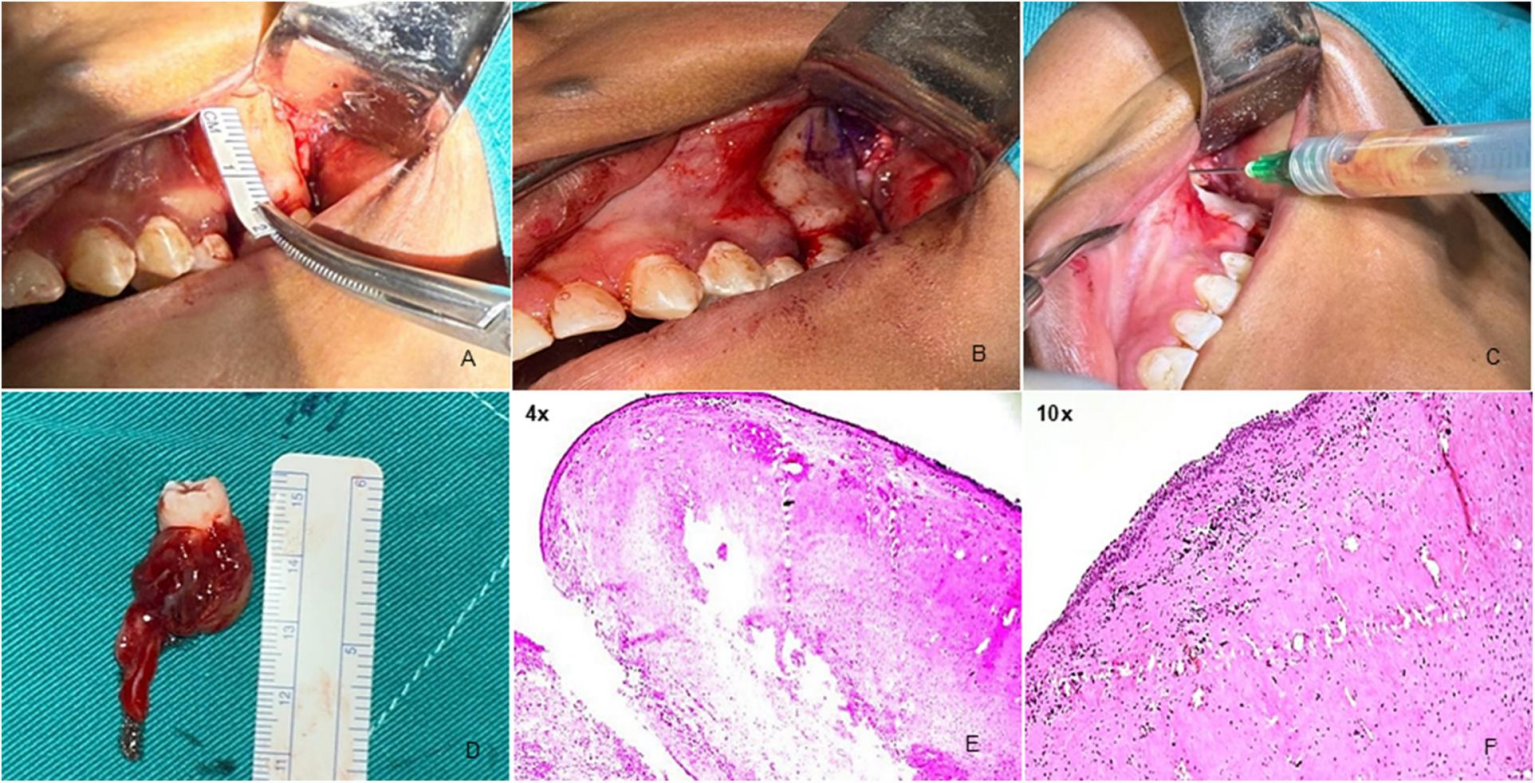
Surgical and histopathological findings. **(A)** Anatomical localization and measurement of 15 mm from the alveolar bone crest to the projection of the tooth on the anterior wall of the maxillary sinus. **(B)** Rectangular bone window measuring 9 mm in height and 16 mm in width. **(C)** Aspiration of the cystic contents. **(D)** Surgical specimen: left maxillary third molar removed together with the cystic capsule immediately after complete enucleation. **(E)** Photomicrograph, hematoxylin and eosin staining, 4× magnification, Nikon Eclipse E100 light microscope. **(F)** Photomicrograph, hematoxylin and eosin staining, 10× magnification, Nikon Eclipse E100 light microscope. A cystic wall lined by non-keratinizing squamous epithelium without parakeratosis, with reactive changes and epithelial erosion, is observed, along with fibroconnective stroma showing a lymphoplasmacytic inflammatory infiltrate with polymorphonuclear leukocytes, foamy histiocytes, bacterial aggregates, and crystals.

The surgical procedure was performed under local anesthesia with 2% lidocaine containing epinephrine 1:80,000. Surgical access was achieved through a Neumann-type vestibular incision extending from the region of the maxillary left second premolar to the distal aspect of the ipsilateral maxillary second molar, followed by elevation of a full-thickness mucoperiosteal flap until the buccal cortical bone was exposed (Figure 2). For anatomical localization of the lesion, a 15-mm distance was measured from the alveolar bone crest to the most superior projection of the dental organ on the anterior wall of the maxillary sinus. A rectangular bone window measuring 9 mm in height and 16 mm in width was outlined and created using a long-shank tapered fissure tungsten carbide bur (FG 701), with copious irrigation using physiological saline solution (0.9% NaCl). Once the bone plate was removed, the fibrous capsule containing the impacted dental organ was exposed; the cystic contents were aspirated, and careful enucleation of the cystic capsule was performed, avoiding dissemination of residual fragments into the maxillary sinus. Subsequently, the surgical bed was irrigated profusely, hemostasis was achieved, and layered closure was performed with 4-0 absorbable polyglycolic acid sutures (Vicryl®).

The specimens obtained were submitted for histopathological examination to confirm the differential diagnosis among dentigerous cyst, unicystic ameloblastoma, and odontogenic keratocyst. The histopathological report described a cystic wall lined by a non-keratinising squamous epithelium, without parakeratosis, with reactive changes, erosion of the fibroconnective stroma and a lymphoplasmacytic inflammatory infiltrate with polymorphonuclear cells, foamy histiocytes, bacterial aggregates, and crystals; the lesion was negative for malignancy and compatible with a dentigerous cyst (Figure 2).

**Figure 3:**
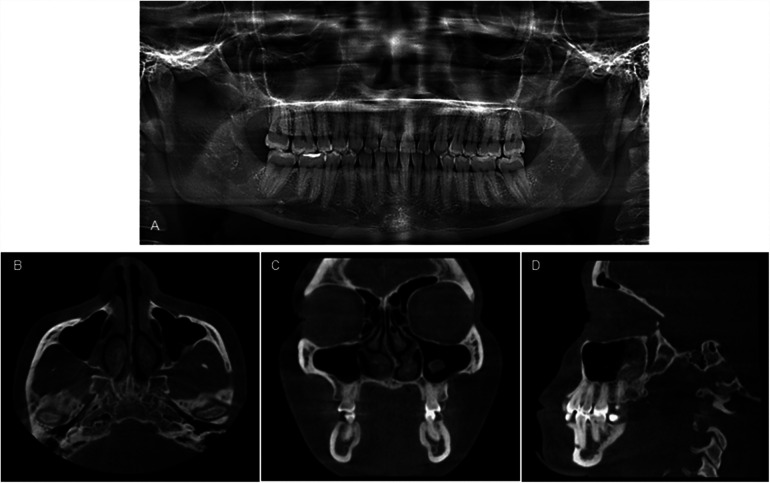
Six-month follow-up imaging. **(A)** Follow-up orthopantomography showing the left maxillary sinus with no evidence of pathological involvement. Follow-up cone-beam computed tomography (CBCT): **(B)** axial, **(C)** coronal, and **(D)** sagittal views showing a well-defined residual hypodense area compatible with postoperative bone remodeling, with no imaging evidence of recurrence of the dentigerous cyst in the left maxillary sinus.

**Figure 4:**
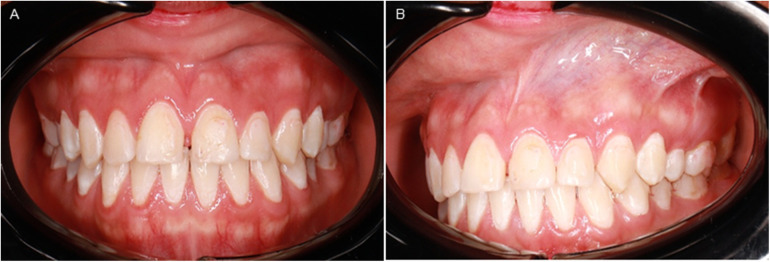
Two intraoral photographs obtained at the six-month postoperative follow-up. Panel A shows a frontal view of the maxillary and mandibular teeth and gingival tissues with the mouth open. Panel B shows a left lateral intraoral view focused on the previously treated maxillary surgical region. Both panels demonstrate complete mucosal healing and adequate repositioning of the soft tissues. The surgical area appears closed and stable, without visible dehiscence or clinical signs of infection.

Postoperative outpatient pharmacological therapy included amoxicillin–clavulanic acid 1 g every 12 h for 7 days, paracetamol 1 g every 8 h for 5 days, ibuprofen 400 mg every 8 h for 4 days, oxymetazoline two sprays in each nostril every 12 h for 4 days, loratadine 10 mg once daily for 5 days and intermittent local cold application for 48 h. Periodic postoperative clinical and imaging follow-up visits were maintained. At 7 days, mild edema was observed in the left middle facial third, with the surgical wound showing active healing and intact sutures. At one month, there were no signs of infection or dehiscence and adequate mucosal healing was observed; during this same appointment, the remaining third molars were extracted. At six months, the patient reported no symptoms, observed an improvement in nasal patency, and complete resolution of the postnasal drip sensation; CBCT imaging showed a well-defined residual hypodense area compatible with postoperative bone remodeling, with no imaging evidence of recurrence of the dentigerous cyst in the left maxillary sinus (Figure 3). Intraoral examination revealed complete mucosal healing with proper repositioning of soft tissues in the surgical area, with no signs of dehiscence or infection (Figure 4, Table 1).

**Table 1:** Clinical timeline of the case report.

Clinical stage	Description
Medical consultation	A 22-year-old female patient presented with a four-month history of nasal congestion and postnasal drip sensation, without a relevant medical history.
Imaging assessment	Orthopantomography: radiolucent image associated with the crown and root of the left maxillary third molar displaced into the ipsilateral maxillary sinus. Cone-beam computed tomography (CBCT): ectopic left maxillary third molar associated with a well-defined hypodense image (≈ 3.3 × 2.0 × 1.9 cm) compatible with a cystic lesion.
Diagnosis	Differential diagnosis: dentigerous cyst, unicystic ameloblastoma, or odontogenic keratocyst. Definitive diagnosis: dentigerous cyst.
Treatment	Complete cystic capsule enucleation and odontectomy of the ectopic left third maxillary molar were performed using the Caldwell–Luc approach under local anesthesia.
Follow-up	Postoperative clinical and imaging (CBCT) follow-up at 7 days, 30 days, and 6 months.
Outcomes	The patient was asymptomatic, without sinonasal symptoms. CBCT imaging showed a well-defined residual hypodense area compatible with postoperative bone remodeling, with no evidence of recurrence in the maxillary sinus at six months of follow-up.

From the patient’s perspective, she experienced progressive improvement in nasal breathing and complete resolution of the postnasal drip sensation during follow-up and expressed satisfaction with the surgical result.

## 3 Discussion

The present case describes the treatment of a dentigerous cyst associated with an ectopic third molar in the maxillary sinus using the Caldwell–Luc technique guided by cone-beam computed tomography (CBCT) and performed under local anesthesia; this therapeutic approach was based on the size and location of the lesion, which are determining criteria for surgical planning (9, 10).

Regarding the etiology of this condition, the ectopic displacement of maxillary third molars into the maxillary sinus is considered a multifactorial phenomenon, in which progressive expansion of a dentigerous cyst acts as a primary driver by exerting pressure on surrounding bone structures and facilitating migration of the tooth into the sinus cavity (11). Additional mechanisms include developmental disturbances during odontogenesis, aberrant eruption pathways, and anatomical constraints of the posterior maxilla, which may contribute to the position of ectopic teeth individually or in combination (3).

Considering the anatomical complexity of this displacement, CBCT proved essential for preoperative evaluation, as it allowed for a precise, three-dimensional assessment of the extent of the lesion and its spatial relationship to the maxillary sinus and adjacent anatomical structures, which directly influenced surgical planning (6).

The lesion appeared in CBCT as a unilocular hypodense image with well-defined borders, associated with the ectopic left maxillary third molar, causing cortical expansion, thinning of the bony walls, and partial occupation of the left maxillary sinus. These imaging findings are consistent with the typical radiological features of dentigerous cysts and contributed to narrowing the differential diagnosis and planning the surgical approach (6, 12).

While these tomographic findings provided a clear direction, it is important to consider odontogenic keratocyst in the differential diagnosis because of its aggressive behavior and higher recurrence rate compared with dentigerous cysts, highlighting the importance of long-term follow-up (13–16). Unicystic ameloblastoma should also be considered due to its similar appearance. In addition, selected maxillary sinus lesions may present with comparable clinical and imaging features. Therefore, histopathological examination remains essential for establishing a definitive diagnosis (4, 5). This diagnostic certainty is critical, as it directly dictates the therapeutic approach; for instance, while Carnoy's solution has been proposed as an adjunctive treatment for odontogenic keratocysts because of their higher recurrence potential, its use was not indicated in the present case since histopathological examination confirmed a dentigerous cyst (16).

Currently, there is no universal consensus on the ideal surgical approach for managing cystic lesions in the maxillary sinus. However, the Caldwell–Luc approach has historically been considered the standard because it provides wide access that ensures complete removal of the lesion (10). In recent years, transnasal functional endoscopic sinus surgery has gained increasing relevance as a minimally invasive alternative, with lower morbidity and faster recovery (15, 17). Recent literature reports that endoscopic removal of ectopic maxillary sinus teeth associated with dentigerous cysts can be effective in selected cases; however, surgical access may be challenging in large lesions or when the ectopic tooth is deeply positioned (18). A systematic review of endoscopic removal of ectopic sinonasal teeth similarly noted that, although endoscopic approaches have become increasingly utilized in recent years, the Caldwell–Luc technique has classically been the standard for such cases, underscoring that surgical decision-making should be individualized according to lesion characteristics (19).

Therefore, given that the lesion was approximately 3.3 cm in its greatest dimension and partially occupied the maxillary antrum, and that the ectopic third molar was in a deep position, the Caldwell–Luc approach was preferred. This approach provides direct visualization and optimal access to complete cystic capsule enucleation (3). This decision minimized the risk of leaving residual fragments within the sinus, which is a documented limitation of the endoscopic approach, particularly in large lesions or those with complex anatomical access (3). In contrast, the Caldwell–Luc technique remains a highly relevant and accessible alternative, whereas the endoscopic approach requires highly specialized equipment, which is a significant limitation in resources-constrained settings, such as most public hospitals. In this case, performing the procedure under local anesthesia may have contributed to reducing hospital costs and optimizing the use of surgical resources. Although this approach carries inherent risks such as infraorbital hypoesthesia or the development of an oroantral fistula (2), the literature supports its use in situations where complete enucleation of the pathology takes precedence over invasiveness, findings consistent with those reported by Thakur et al. (11) and Torres Restrepo et al. (12), who support this criterion in similar contexts. Despite the inherent risks of the method, none of the aforementioned complications occurred. Removal under direct visualization, facilitated by the defined bone window and careful enucleation, resulted in complete enucleation of the pathology, with subsequent complete resolution of the sinonasal symptoms.

One of the main strengths of the present report was the preoperative use of CBCT, which not only enabled precise surgical planning and contributed to a favorable clinical outcome with complete resolution of sinonasal symptoms, but also allowed for improved characterization and enhanced diagnostic accuracy of dentigerous cysts with variable radiological presentations (14). These findings also contribute to the existing literature by re-evaluating a classic surgical method performed entirely under local anesthesia, supporting the continued clinical relevance of the Caldwell–Luc approach as a predictable and accessible treatment option for selected extensive maxillary sinus lesions, particularly in resource-limited settings (10, 15).

Despite the favorable outcome, the inherent limitations of this report should be acknowledged. The generalizability of the findings is limited, as this is a single case. Although recurrence rates for dentigerous cysts are generally low after complete enucleation, long-term follow-up remains advisable (1, 4). A further limitation is the follow-up duration: the six-month follow-up presented in this report should be interpreted as a short-term clinical outcome, and because odontogenic cystic lesions may require longer observation to reliably rule out recurrence, longer follow-up periods are necessary to definitively exclude it.

This clinical case highlights that the Caldwell–Luc approach remains a viable and effective option for complete enucleation of a dentigerous cyst associated with an ectopic third molar in the maxillary sinus, especially when the location and size of the lesion may compromise the reliability of minimally invasive techniques.

Therefore, future research should focus on multicenter comparative studies or case series evaluating both techniques: the Caldwell–Luc approach and functional transnasal endoscopic sinus surgery. This would allow for an assessment of the effectiveness of each approach in terms of complete enucleation rate, postoperative morbidity, and long-term outcomes, including the rate of recurrence. Such studies would be essential to guide evidence-based clinical decision-making and to establish the cost-effectiveness of endoscopic equipment in different healthcare settings.

## Data Availability

All relevant data supporting the findings of this study are contained within the article. No additional datasets were generated or analyzed for this study.
